# Supplementary material for: All three MutL complexes are required for repeat expansion in a human stem cell model of CAG-repeat expansion mediated glutaminase deficiency
Source: bioRxiv. 2024 May 9:2023.12.26.573357. Preprint. [Version 3] doi: 10.1101/2023.12.26.573357 (PMC10802475; doi:10.1101/2023.12.26.573357)

bioRxiv preprint doi: https://doi.org/10.1101/2023.12.26.573357; this version posted May 9, 2024. The copyright holder for this preprint (which was not certified by peer review) is the author/funder. This article is a US Government work. It is not subject to copyright under 17 USC 105 and is also made available for use under a CC0 license.

Supplementary Methods

Western blotting

To prepare total cell lysates, cells were pelleted at 250 x g for 5 min and washed once with ice-cold PBS supplemented with 1X protease inhibitor cocktail (Sigma-Aldrich, St. Louis, MO, P8340). The cell pellet was then resuspended in the lysis buffer (10 mM Tris Cl pH 7.5, 1 mM EDTA pH 8.0, 1% Triton X-100, 1X protease inhibitor cocktail) and incubated on ice for 10 min followed by sonication (3 cycles of 30 seconds ON/30 seconds OFF) at medium setting using Bioruptor® (Diagenode, Denville, NJ) to solubilize proteins and shear the DNA. The protein amount was quantified using Bio-Rad Protein Assay Dye Reagent Concentrate (Bio-Rad Laboratories, Inc., Hercules, CA, #5000006) as per manufacturer's protocol. Before using the lysate for western blot analyses, 1X volumes of Novex® LDS Sample Buffer (Thermo Fisher Scientific, Waltham, MA, NP0007) and NuPAGE® Sample Reducing Agent (Thermo Fisher Scientific, NP0009) were added, and the samples were heated at 75 °C for 10 min.

Twenty (for PMS1) or thirty (for PMS2) micrograms of total cell lysates were run on NuPAGE™ 4-12% Bis-Tris gel (Thermo Fisher Scientific, NP0322BOX) and transferred to nitrocellulose membrane using the Trans-Blot Turbo RTA mini 0.2 µm nitrocellulose transfer kit (1704270) and Trans-Blot® Turbo™ Transfer system from Bio-Rad Laboratories. The membrane was blocked for two hours with 5% blocking agent (GE Healthcare Bio-Sciences, Pittsburg, PA, RPN2125) in TBST (1X Tris buffered saline with 0.1% Tween 20). The following antibodies were used, 1:1000 diluted anti-PMS1 rabbit monoclonal antibody [EPR27158-78] (Abcam, ab315798) overnight at 4°C, 1:500 diluted anti-PMS2(B3) mouse monoclonal antibody (Santa Cruz Biotechnology, # sc-25315) overnight at 4°C, 1:10,000 diluted anti-b-actin mouse monoclonal antibody (Invitrogen # MA1-140) for 1 hour at room temperature. Following incubation with the primary antibodies, the blots were washed three times with TBST for 5 minutes each and then probed with either 1:2000 diluted HRP-labeled secondary Rabbit antibody (Millipore Sigma, (GENA934) or 1:5000 diluted HRP-labeled secondary mouse antibody (Millipore Sigma (12-349) for 1 hour. The blot was then washed three times with TBST for 5 minutes each and once with TBS. The signal was detected using ECL™ Prime detection reagents (GE Healthcare Bio-Sciences) and imaged with ChemiDoc imaging system (Bio-Rad Laboratories).

bioRxiv preprint doi: https://doi.org/10.1101/2023.12.26.573357; this version posted May 9, 2024. The copyright holder for this preprint (which was not certified by peer review) is the author/funder. This article is a US Government work. It is not subject to copyright under 17 USC 105 and is also made available for use under a CC0 license.

Supplementary Figures

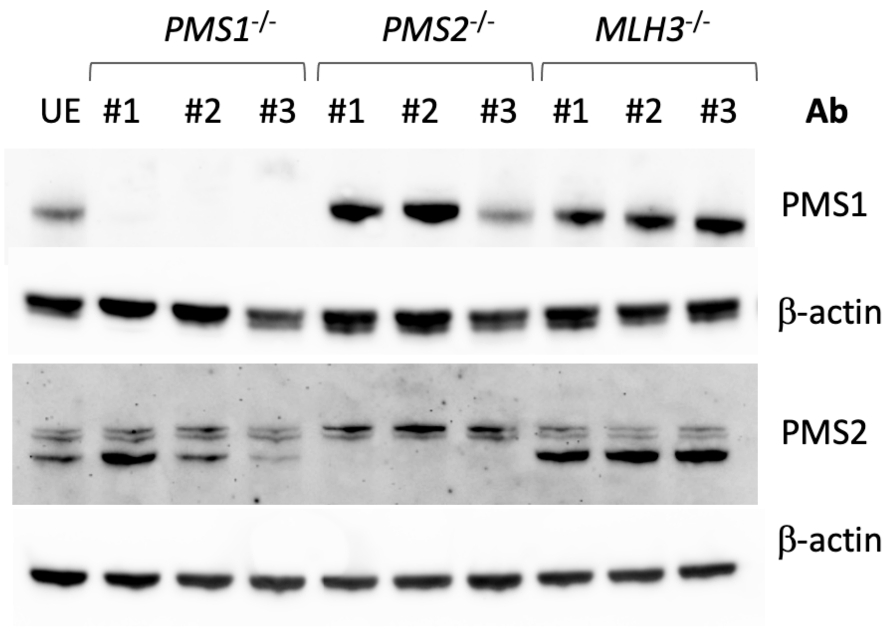

Fig. S1. Analysis of MLH1-binding proteins in PMS1, PMS2 and MLH3 gene edited lines. a) Western blots of the unedited (UE) GLSD patient iPSC line and the *PMS1*, *PMS2* and *MLH3* null lines derived from it used in this study challenged with antibodies for PMS1, PMS2 and β-actin.

bioRxiv preprint doi: https://doi.org/10.1101/2023.12.26.573357; this version posted May 9, 2024. The copyright holder for this preprint (which was not certified by peer review) is the author/funder. This article is a US Government work. It is not subject to copyright under 17 USC 105 and is also made available for use under a CC0 license.

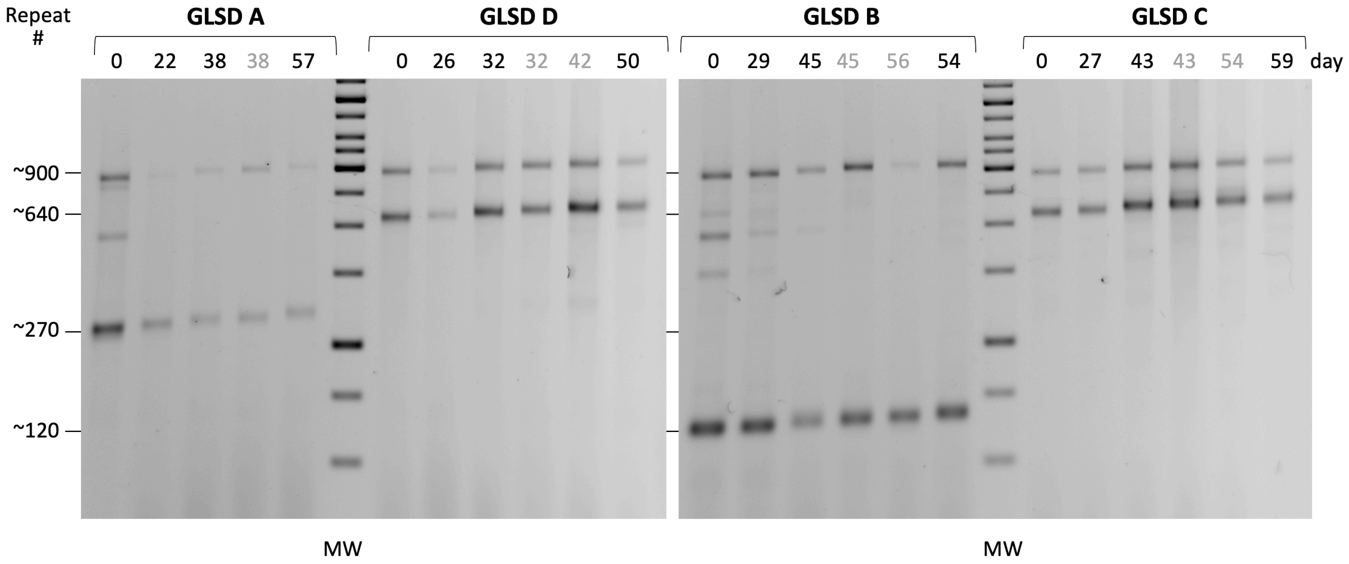

Fig. S2. Expansion of the CAG-repeat tract over time in 4 different patient iPSC lines. The iPSC lines were propagated in culture as described in the Materials and Methods section and DNA isolated from the lines at different time points as indicated. The PCR products obtained by amplification through the GLS repeat in iPSCs derived from a patient (Patient 2 [1]) were then resolved by agarose gel electrophoresis alongside a GeneRuler 1 kb DNA ladder (Thermo Fisher Scientific, SM0311). The approximate repeat numbers in different starting alleles are indicated alongside. The samples shown in grey font were obtained from cultures that had been grown at 38.5 ˚C for 6 days prior to harvesting to eliminate residual Sendai virus. Interestingly, they show a slightly more expansion than the cells maintained at 37˚C which may point to an effect of temperature on expansion rate.

bioRxiv preprint doi: https://doi.org/10.1101/2023.12.26.573357; this version posted May 9, 2024. The copyright holder for this preprint (which was not certified by peer review) is the author/funder. This article is a US Government work. It is not subject to copyright under 17 USC 105 and is also made available for use under a CC0 license.

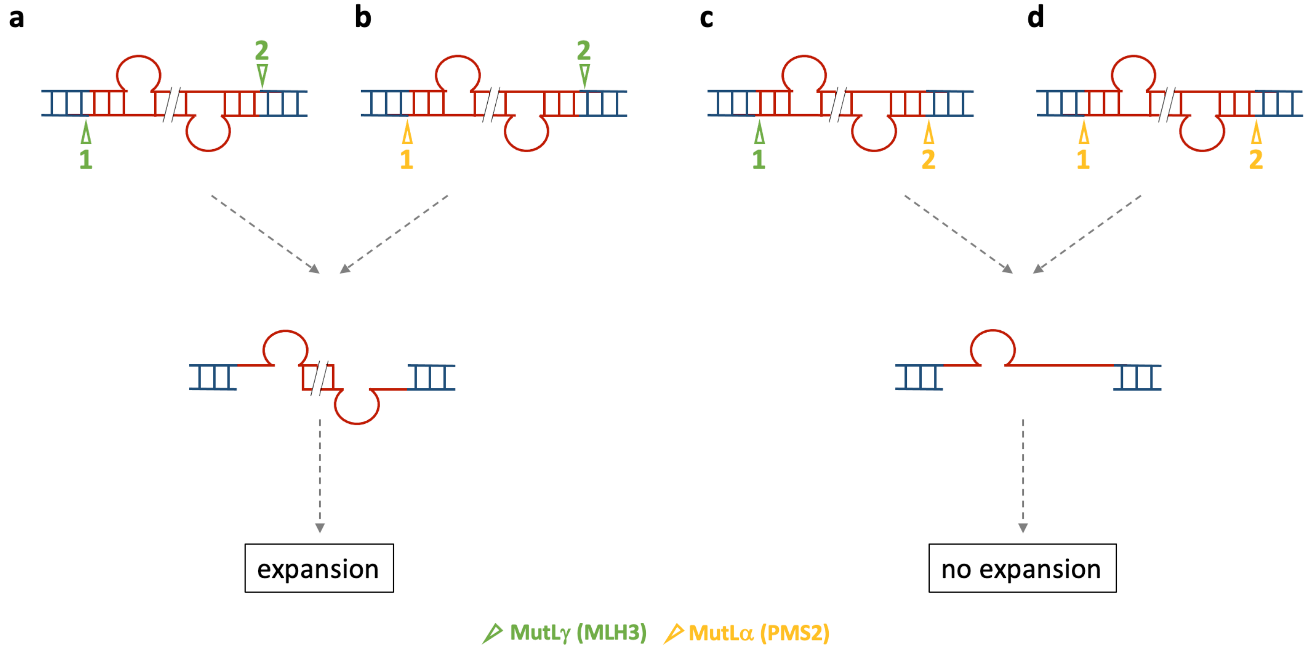

Fig. S3. Model for PMS2's promutagenic and antimutagenic roles in repeat expansion. In this model, the substrate for expansion is a double loop-out structure formed in the repeat. Formation of the substrate during transcription may explain the transcriptional dependence [2]. It may also explain the high frequency with which expansion occurs. The size of the loop-outs determines the net gain of repeats and thus the size of the expansion. Since some expansion-prone repeats do not form hairpins and most expansions involve the addition of a single repeat, a loop-out of a single repeat is shown. However, it may be that larger loop-outs form, with or without hairpin formation, and this could contribute to the small number of larger expansions that are occasionally seen. Since PMS1 has no nucleolytic activity [3], it is presumed to have a structural role in expansion that is not shown in this model. Each loop-out is bound by MutLγ or MutLα and the binding depending on some combination of the relative abundance of the two complexes and their relative binding affinities. Each MutL protein then makes a cut on one or other strand, depending on their cleavage preferences, with MutLγ cutting the strand opposite the loop-out and MutLα showing no bias for which strand is cut when there is no pre-existing nick and cutting on the nicked strand when there is [4,5]. Each possible set of cuts is shown assuming that the first cut (cut 1) is performed by the MutL complex bound to the bottom strand loop-out. When both cuts (cuts 1 and 2) are made by MutLγ (a), an intermediate containing gaps opposite each loop-out is produced. Secondary structures formed by the looped-out bases might limit further resection that would otherwise result in the loss of one or both of the looped-out regions. Filling of both gaps by a gap-filling polymerase and ligation results in an expansion. Gap-filling of each loop-out adds a total of 3 bases on each strand, resulting in a net gain of one repeat. If MutLα makes the first cut and it occurs on the strand opposite the loop-out to which it is bound (b) and there is enough MutLγ to make the second cut, then it too will result in an expansion. On

bioRxiv preprint doi: https://doi.org/10.1101/2023.12.26.573357; this version posted May 9, 2024. The copyright holder for this preprint (which was not certified by peer review) is the author/funder. This article is a US Government work. It is not subject to copyright under 17 USC 105 and is also made available for use under a CC0 license.

the other hand, if MutLγ makes the first cut and MutLα the second (c), or if MutLα makes both cuts (d) this would result in two cuts on the same strand. Repair synthesis in any of these cases would then restore the original allele. Thus, in cells where MutLγ is sufficient to process all the expansion substrates (a), they would all be processed to generate expansions. When MutLα predominates, all substrates would be processed so as to restore the original allele (d), and when MutLγ and MutLα are both involved in processing the expansion substrate, some would be processed to generate expansions (b) and some fraction would be processed so as to restore the original allele (c) Since the expansion rate depends on the rate at which the substrate forms and is processed, rates that would be shared amongst all cells of a given type, all cells in a homogenous population of cells would expand at a similar rate. This results in a similar shift of most of the population to progressively larger alleles with time.

Supplementary References

1 van Kuilenburg, A. B. P. *et al.* Glutaminase Deficiency Caused by Short Tandem Repeat Expansion in GLS. *N Engl J Med* **380**, 1433-1441 (2019). https://doi.org:10.1056/NEJMoa1806627

2 Lokanga, A. R., Zhao, X. N., Entezam, A. & Usdin, K. X inactivation plays a major role in the gender bias in somatic expansion in a mouse model of the fragile X-related disorders: implications for the mechanism of repeat expansion. *Hum Mol Genet* **23**, 4985-4994 (2014). https://doi.org:10.1093/hmg/ddu213

3 Cannavo, E., Gerrits, B., Marra, G., Schlapbach, R. & Jiricny, J. Characterization of the interactome of the human MutL homologues MLH1, PMS1, and PMS2. *J Biol Chem* **282**, 2976-2986 (2007). https://doi.org:10.1074/jbc.M609989200

4 Pluciennik, A. *et al.* PCNA function in the activation and strand direction of MutLalpha endonuclease in mismatch repair. *Proc Natl Acad Sci U S A* **107**, 16066-16071 (2010). https://doi.org:10.1073/pnas.1010662107

5 Kadyrova, L. Y., Gujar, V., Burdett, V., Modrich, P. L. & Kadyrov, F. A. Human MutLgamma, the MLH1-MLH3 heterodimer, is an endonuclease that promotes DNA expansion. *Proc Natl Acad Sci U S A* **117**, 3535-3542 (2020). https://doi.org:10.1073/pnas.1914718117

bioRxiv preprint doi: https://doi.org/10.1101/2023.12.26.573357; this version posted May 9, 2024. The copyright holder for this preprint (which was not certified by peer review) is the author/funder. This article is a US Government work. It is not subject to copyright under 17 USC 105 and is also made available for use under a CC0 license.

Original Images

Fig. S1

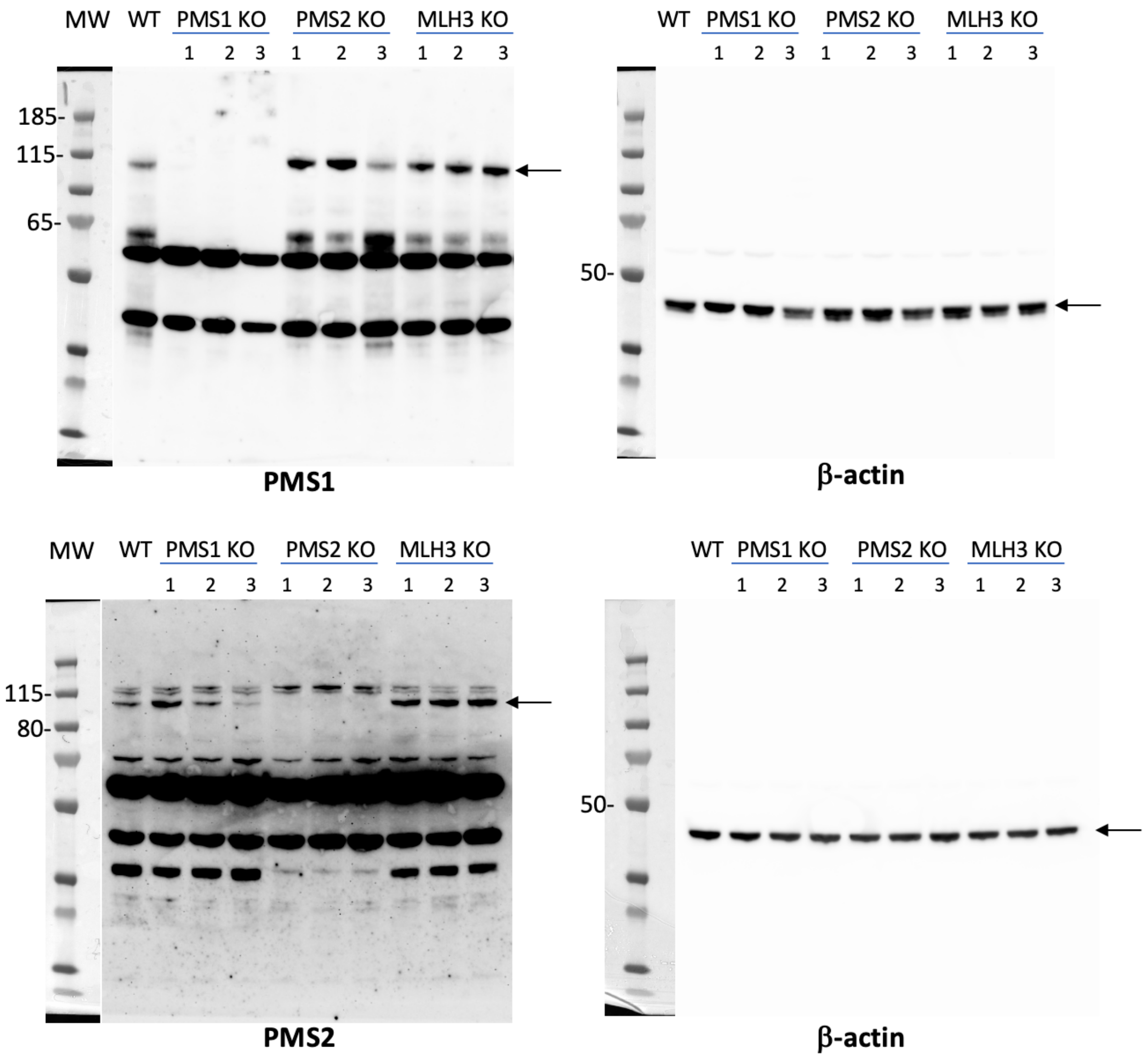

bioRxiv preprint doi: https://doi.org/10.1101/2023.12.26.573357; this version posted May 9, 2024. The copyright holder for this preprint (which was not certified by peer review) is the author/funder. This article is a US Government work. It is not subject to copyright under 17 USC 105 and is also made available for use under a CC0 license.

Fig. S2

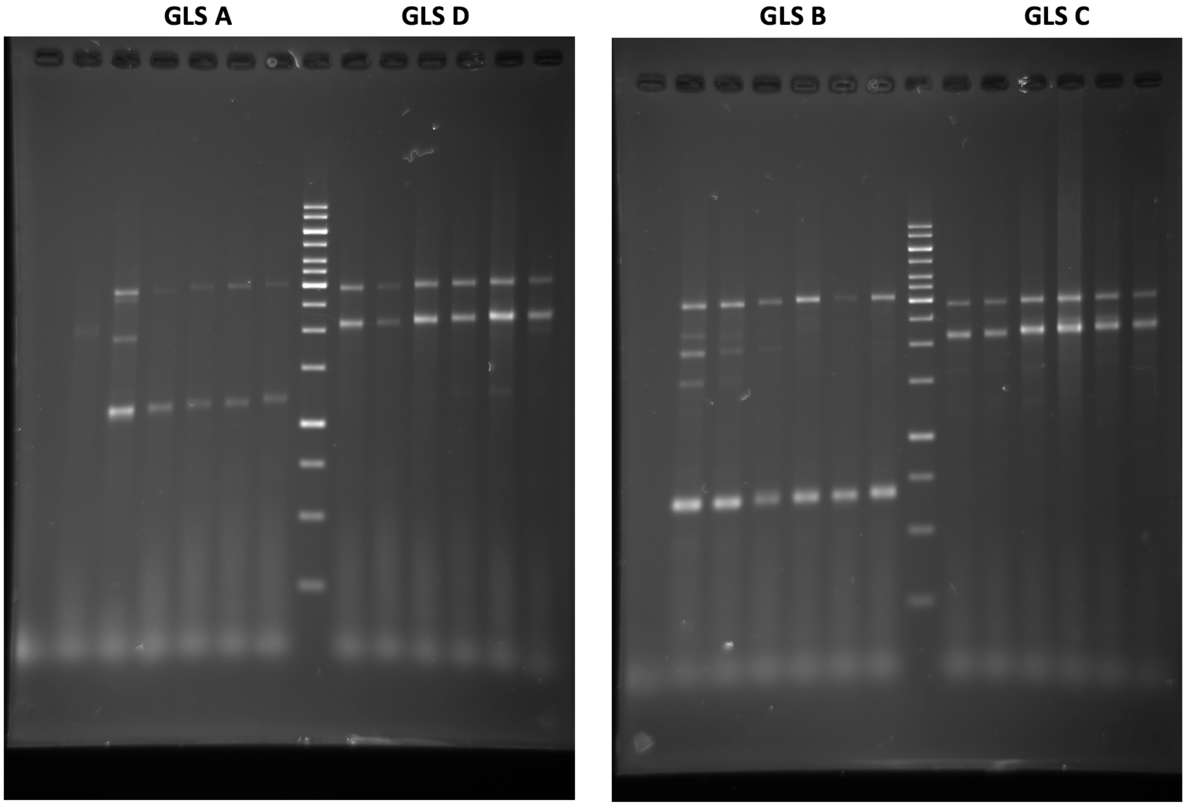

Supplement: Supplement 1 [file NIHPP2023.12.26.573357v3-supplement-1.pdf]
