# Supplementary material for: All three MutL complexes are required for repeat expansion in a human stem cell model of CAG-repeat expansion mediated glutaminase deficiency
Source: bioRxiv. 2024 Jan 8:2023.12.26.573357. Preprint. [Version 2] doi: 10.1101/2023.12.26.573357 (PMC10802475; doi:10.1101/2023.12.26.573357)
Supplement: Supplementary file 1 [file media-1.pdf]

Supplementary Figure Legends

Fig. S1. Expansion of the CAG-repeat tract over time in 4 different patient iPSC lines. The iPSC lines were propagated in culture as described in the Materials and Methods section and DNA isolated from the lines at different passage numbers as indicated. The PCR products obtained by amplification through the GLS repeat in iPSCs derived from a patient (Patient 2 (1)) were then resolved by agarose gel electrophoresis. The approximate repeat numbers of different alleles are indicated alongside. The samples shown in grey font were obtained from cultures that had been grown at 38.5 ˚C for 6 days prior to harvesting to eliminate residual Sendai virus. Interestingly, they show a slightly more expansion than the cells maintained at 37˚C which may point to an effect of temperature on expansion rate.

Fig. S2. Model for PMS2's promutagenic and antimutagenic roles in repeat expansion. In this model, the substrate for expansion is a double loop-out structure formed in the repeat, perhaps during transcription (34). Since PMS1 has no nucleolytic activity (35), it is presumed to have a structural role in expansion that is not shown in this model. Each loop-out is bound by MutLγ or MutLα and the binding frequency of each complex depends on the relative abundance of the two complexes. Each MutL protein then makes a cut on one or other strand. Each possible set of cuts is shown assuming that the first cut (cut 1) is performed by the MutL complex bound to the bottom strand loop-out. When both cuts (cuts 1 and 2) are made by MutLγ (a), an intermediate containing gaps opposite each loop-out is produced. Secondary structures formed by the looped-out bases might limit further resection that would otherwise result in the loss of one or both of

the looped-out regions. Filling of both gaps by a gap-filling polymerase and ligation results in an expansion. If MutLα makes the first cut and it occurs on the strand opposite the loop-out to which it is bound (b) and there is enough MutLγ to make the second cut, then it too will result in an expansion. If, on the other hand, MutLα cuts on the bottom strand (c), and MutLγ makes the second cut, this would result in two cuts on the same strand. A similar result arises if MutLγ makes the first cut and MutLα then cuts on the top strand (d) and when MutLα performs both cuts (e). Repair synthesis in any of these cases would then restore the original allele. Thus, in cells where MutLγ is sufficient to process all the expansion substrates (a), they would all be processed to generate expansions. When MutLα predominates, all substrates would be processed so as to restore the original allele (e), and when MutLγ and MutLα are both involved in processing the expansion substrate, some would be processed to generate expansions and some fraction would be processed so as to restore the original allele.

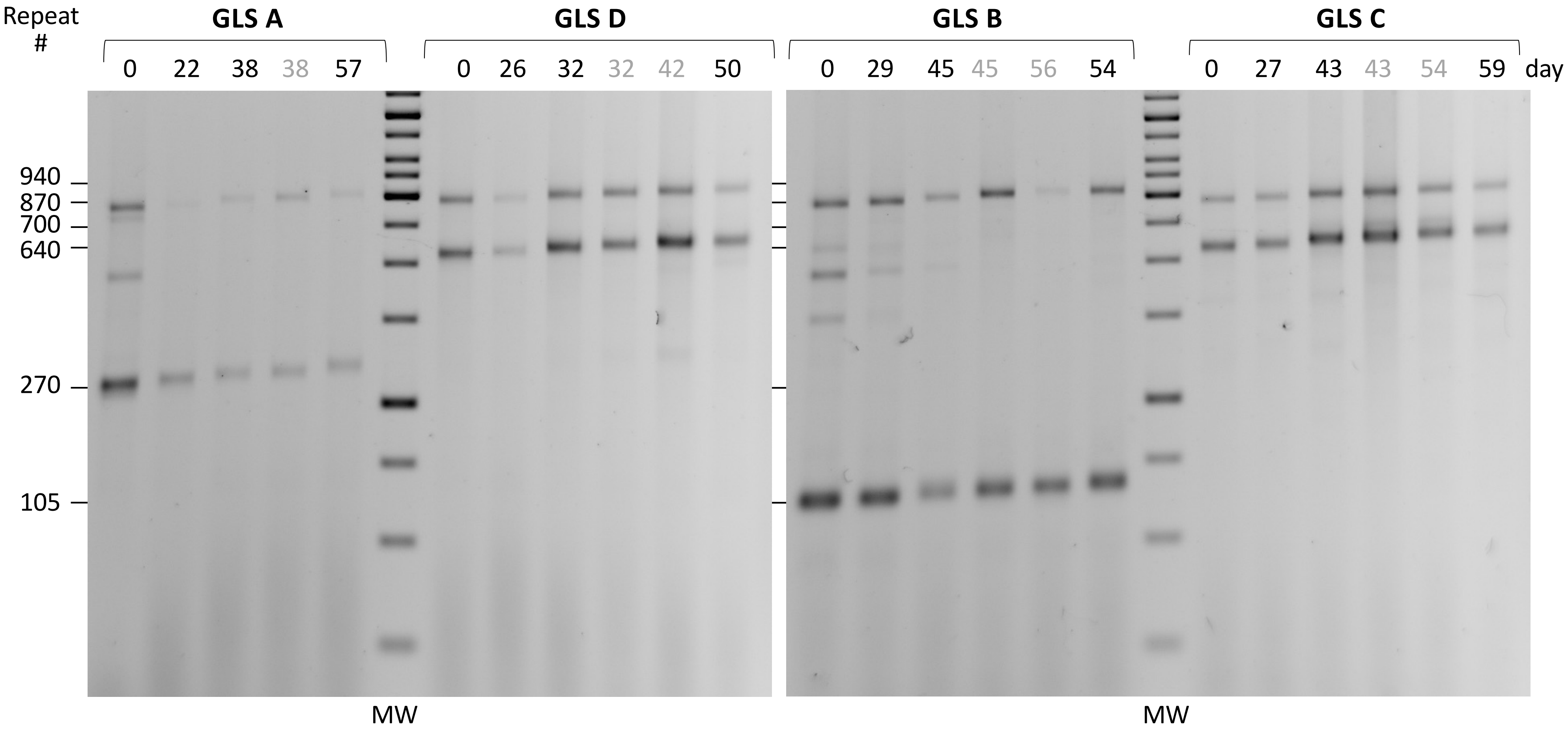
Repeat #
GLS A
GLS D
GLS B
GLS C
0 22 38 38 57
0 26 32 32 42 50
0 29 45 45 56 54
0 27 43 43 54 59
day
940
870
700
640
270
105
MW
MW

Fig. S1

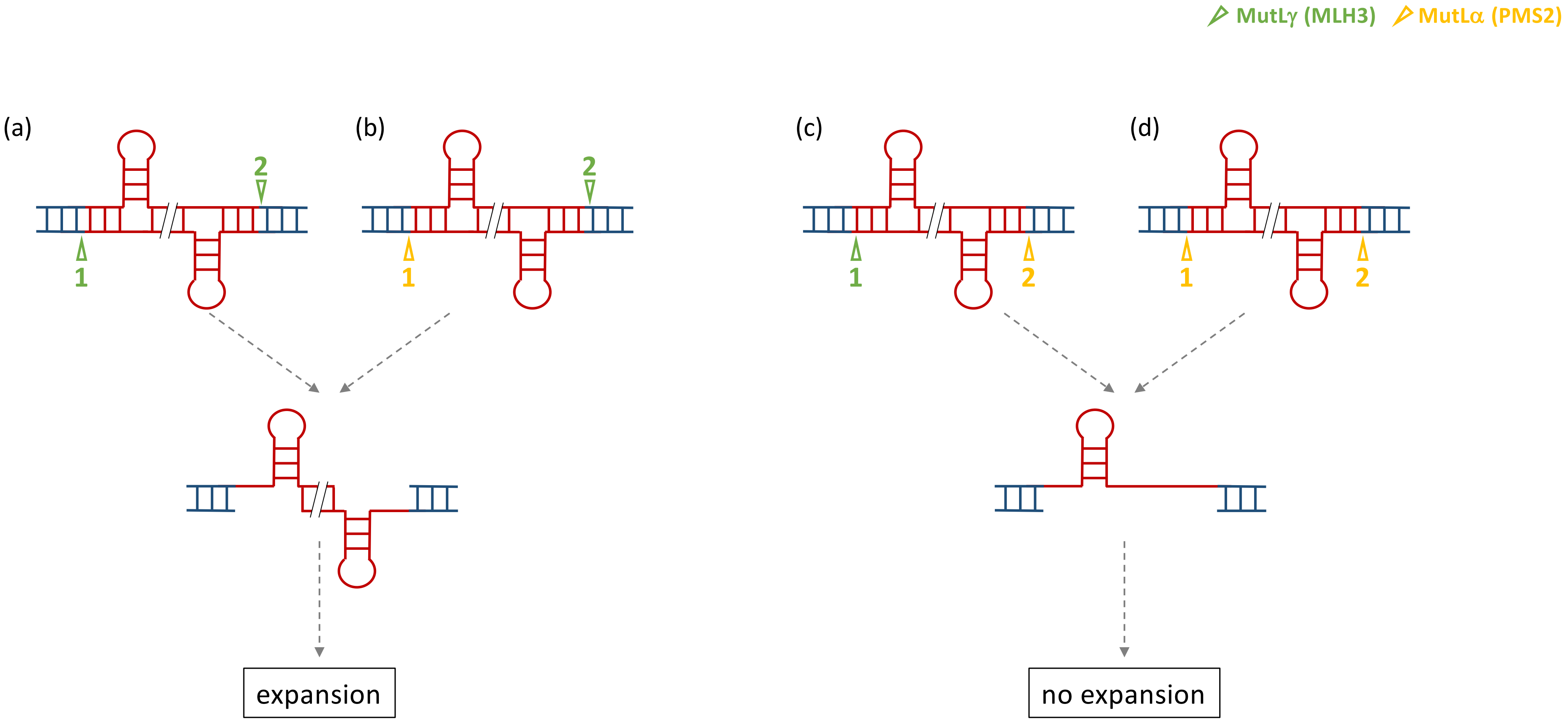
MutLγ (MLH3)
MutLα (PMS2)
(a)
2
1
(b)
2
1
(c)
1
2
(d)
1
2
expansion
no expansion

Fig. S2
